# Laparoscopic Removal of Modified Vertical Uterine Compression Sutures due to Postoperative Focal Pain

**DOI:** 10.1055/s-0040-1708865

**Published:** 2020-03-31

**Authors:** Toshifumi Suzuki, Jun Takeda, Makoto Jinushi, Rie Seyama, Yojiro Maruyama, Shintaro Makino, Mari Kitade, Atsuo Itakura

**Affiliations:** 1 Department of Obstetrics and Gynecology, Juntendo University Faculty of Medicine, Tokyo, Japan

**Keywords:** hemostasis, intrapartum hemorrhage, uterine compression suture, complications, surgical technique

## Abstract

Previously we reported laparoscopic removal of compression sutures due to uterine ischemia and related pain, which has two of the difficult aspects: (1) maneuvering the curved needle to perform compression suturing in the narrow surgical field, and (2) distinguishing between the threads of the cesarean section wound sutures versus the vertical compression sutures during removal, as the threads are the same white color. We performed vertical compression sutures for intrapartum hemorrhage with total placental previa, and modified both the needle type and the color of the thread used for uterine compression sutures during cesarean section. After the operation, we performed successful laparoscopic removal of compression sutures for postoperative focal pain. Changing the needle type and color helped to perform operations. The present case supports the concept that the laparoscopic removal of uterine compression suturing is useful for controlling pain in cases where general analgesics are ineffective.

Uterine compression suturing is a relatively simple and effective method used to manage intrapartum hemorrhage during cesarean section. However, reported complications include abdominal pain, ischemia, and necrosis. 1 2 Postoperatively, it can be difficult to control local pain corresponding to the compression sutures, and general analgesics such as nonsteroidal anti-inflammatory drugs and acetaminophen are ineffective in some cases. In such cases, postoperative pain control via removal of the uterine compression sutures must be considered. We previously reported the laparoscopic removal of vertical uterine compression sutures. 3 However, during the removal procedure, it is difficult to distinguish between the threads of the cesarean section wound sutures versus the compression sutures, as the threads are of the same color. Therefore, we changed the uterine compression suture thread color to violet. In addition, we placed the compression sutures using a straight needle rather than a curved one to increase the handling ease. Recently, a retrospective study, in which novel dedicated blunt straight needle and sutures for uterine compression were used, was reported. 4 Herein, we describe a case of laparoscopic removal of modified vertical uterine compression sutures due to postoperative local pain, and discuss the needle type, suturing, and focal pain control for compression sutures together with the previously reported study.

## Case Report

**Fig. 1:**
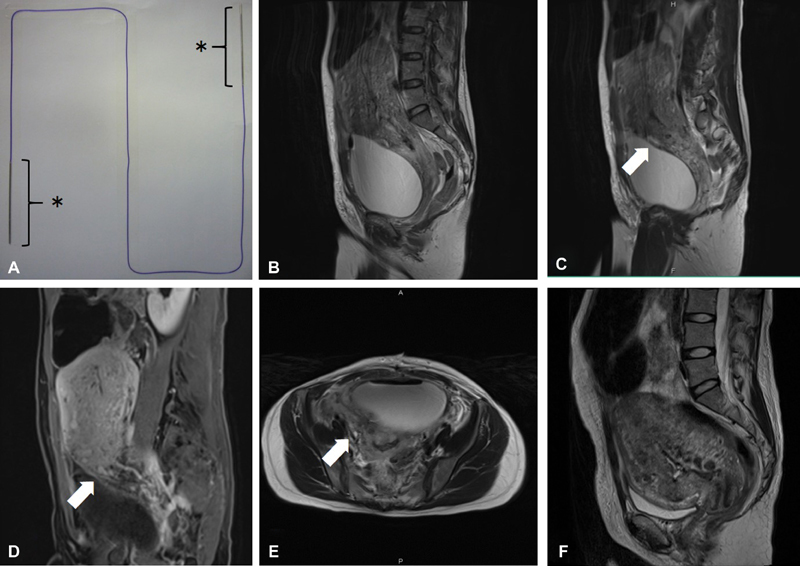
( **a** ) MONODIOX with dull, straight needles. Needle length is 80 mm. Thread length is 70 cm. *indicates the straight dull needles. (b– **e** ) Contrast-enhanced magnetic resonance imaging after vertical compression sutures and the ligation of the right descending uterine artery. ( **f** ) Contrast-enhanced magnetic resonance imaging after removing both vertical compression sutures and the suture of the right descending uterine artery. ( **b** ) T2-weighted sagittal image obtained after vertical uterine compression suturing. ( **c** ) T2-weighted sagittal image obtained with right side of the uterine after vertical uterine compression suturing. Arrow indicates a small hematoma with high intensity surrounding the ligation of the right descending uterine artery and/or right vertical compression suture. ( **d** ) Fat-suppression T1-weighted dynamic magnetic resonance imaging. Arrow indicates a small hematoma. ( **e** ) T2-weighted axial image obtained with right side of the uterine after vertical uterine compression suturing. Arrow indicates a small hematoma with high intensity surrounding the ligation of the right descending uterine artery and/or right vertical compression suture. ( **f** ) T2-weighted sagittal image obtained after removal of vertical uterine compression sutures.

**Fig. 2:**
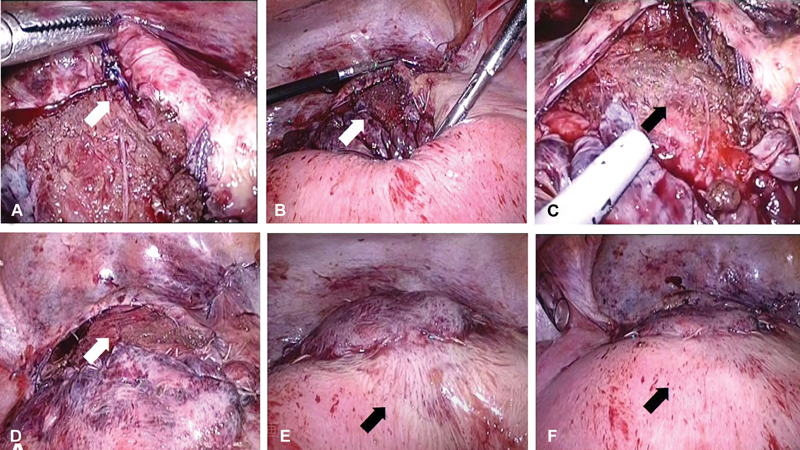
( **a** ) Laparoscopic removal of uterine compression sutures. The right compression suture thread (arrow) was held by laparoscopic forceps. ( **b** – **d** ) Photographs of the lower uterine segment during the laparoscopic surgery. ( **e,f** ) Visual inspection of the anterior uterine wall. ( **b** , **c** ) The lower uterine segment before removal compression sutures. Arrow indicates the lower uterine segment. ( **d** ) The lower uterine segment after removal compression sutures. Arrow indicates the lower uterine segment. ( **e** ) Uterine anterior wall before the removal of vertical uterine compression sutures. Arrow indicates the color of the anterior uterine wall. ( **f** ) Uterine anterior wall after the removal of vertical uterine compression sutures. Arrow indicates the color of the anterior uterine wall.

The patient was a gravida 1, para 0, 36-year-old Japanese woman diagnosed with total placenta previa. An elective cesarean section was performed at 37 weeks and 3 days of gestation. Intrapartum hemorrhage during cesarean section was treated with double vertical uterine compression sutures and intrauterine balloon tamponade. Violet-colored MONODIOX (Alfresa Pharma Corporation, Osaka, Japan) with a straight needle, which is not a dedicated blunt needle, was used for the vertical uterine compression sutures ( Fig. 1a ). Vertical compression suturing was easily performed with the straight needle. Although the bleeding lessened, it still continued, and, therefore, the right descending uterine artery was ligated using white-colored 0 Vicryl plus (Ethicon, Somerville, New Jersey, United States). Blood loss could be controlled by compression sutures and the ligation of the right descending uterine artery. The cesarean section was then finished without blood transfusion, and blood loss was ∼2,100 mL, including amniotic fluid. On postoperative day 0, the patient experienced unbearable continuous pelvic pain with a right predominance with a numerical rating scale (NRS) of 5 points, despite analgesic administration; this pain prevented early postoperative ambulation. Although contrast-enhanced magnetic resonance imaging (MRI) did not clearly reveal uterine ischemia and necrosis, but due to the small hematoma (20 × 5 mm) surrounding the ligation of the right descending uterine artery and/or right vertical compression suture on postoperative day 1, we considered that the local pain was associated with uterine ischemia ( Fig. 1b–e ). Therefore, laparoscopic removal of the compression sutures and uterine artery ligature was performed on postoperative day 2. During the laparoscopic operation, the violet-colored compression suture threads were easily found and removed ( Fig. 2a ). In contrast, the removal of the white-colored suture thread used to ligate the right descending uterine artery required some time. Unexpectedly, after removing both compression sutures and the right descending artery, the color of the lower uterine segment was almost same ( Fig. 2b–d ). However, on intraoperative visual inspection, the color of the anterior uterine wall was pinkish-white to pinkish-red ( Fig. 2e,f ). Postoperatively, the NRS for pain improved from 5 to 2 points, and there was no persistent hemorrhage. On day 2 after laparoscopic surgery, contrast-enhanced MRI was performed. Contrast-enhanced MRI revealed that the size of the uterine corpus had increased in comparison with the preoperative status ( Fig. 1f ). Although there was no clear improvement in uterine ischemia on MRI, the pain had improved and the patient became able to ambulate ( Fig. 1b–f ). The patient had an uneventful postoperative course, and was discharged from hospital 9 days after the cesarean section.

## Discussion

We searched the PubMed database (https://www.ncbi.nlm.nih.gov/pubmed/) for associations between compression suture, needle, and device. To the best of our knowledge, at least four case reports described the use straight needles for uterine compression sutures, but did not discuss the effectiveness of this method, 5 6 7 8 while recently one retrospective study was first reported the use of dedicated blunt straight needles and sutures for uterine compression sutures of modified Hayman suture. 4 Matsuzaki et al used No. 2 Polydioxanone (2-Monodiox), which is novel dedicated blunt straight needles. 4 The retrospective study was suggested that the uterine preservation rate was similar for 2-Monodiox with modified Hayman suture and No.1 poliglecaprone 25 sutures with B-Lynch suture without the occurrence of severe complications. 4 We modified both the needle type and the thread color. As a straight needle could penetrate the anteroposterior uterine wall, care was taken during the needle handling. MONODIOX, which is used for our case, is not dedicated blunt straight needles for uterine compression sutures. Although 2-Monodiox has not been used for uterine vertical compression sutures in previous study, our case together with previous study suggested MONODIOX and 2-Monodiox are useful needles for various uterine and vertical compression sutures. The violet-colored standing thread was easily identified during laparoscopic removal. However, some time was required to identify the white thread used to ligate the right descending uterine artery because of the color similarity of both uterus connective tissue and myometrium suturing threads. Therefore, colors other than white should be used for sutures that could possibly require removal, although it is difficult to simply compare each operation time.

Aboulfalah et al suggested that removal of the uterine compression sutures 24–48 hours after application prevents uterine infection and synechia. 9 In the present case, laparoscopic removal of compression suturing within 48 hours postoperatively improved the pelvic pain that was unresponsive to analgesics, and prompted early postoperative ambulation. In our case, especially, the unbearable continuous pelvic pain with a right predominance occurred after cesarean section. We speculated that the right uterine artery ligation with puerperal involution of the uterus might cause severe postoperative pain. During the laparoscopic surgery, it was noted that the anterior uterine wall had changed to a pinkish-red color. These findings suggest that uterine ischemia was improved by the laparoscopic removal surgery, although this was not confirmed on MRI findings.

Furthermore, Aboulfalah et al reported the removal of nonabsorbable sutures by simple wire traction without any anesthesia after cesarean section. 9 While their method has the advantage of not requiring anesthesia, our method is suitable for any type of uterine compression sutures.

In conclusion, the present case supports the concept that the laparoscopic removal of uterine compression suturing is useful for controlling pain in cases where general analgesics are ineffective. Using a straight needle made the application of compression sutures easier, and using violet-colored thread enabled easier identification of the compression sutures during laparoscopic removal. We recommend using different color threads for the cesarean section wound sutures and the related compression sutures.
